# Resonant Tunneling Induced Enhancement of Electron Field Emission by Ultra-Thin Coatings

**DOI:** 10.1038/s41598-019-43149-y

**Published:** 2019-05-02

**Authors:** Christian Henkel, Robert Zierold, Adithya Kommini, Stefanie Haugg, Chris Thomason, Zlatan Aksamija, Robert H. Blick

**Affiliations:** 1 Present Address: Center for Hybrid Nanostructures (CHyN), Universität Hamburg, Luruper Chaussee 149, 22761 Hamburg, Germany; 2 Electrical and Computer Engineering, University of Massachusetts, 100 Natural Resources Road, Amherst, 01003-9292 MA United States; 3 Materials Science and Engineering, University of Wisconsin-Madison, 1550 University Avenue, Madison, 53706 WI United States; 4 Institute of Experimental Physics, Universität Hamburg, Luruper Chaussee 149, 22761 Hamburg, Germany

**Keywords:** Electronic properties and materials, Surfaces, interfaces and thin films

## Abstract

The emission of electrons from the surface of a material into vacuum depends strongly on the material’s work function, temperature, and the intensity of electric field. The combined effects of these give rise to a multitude of related phenomena, including Fowler-Nordheim tunneling and Schottky emission, which, in turn, enable several families of devices, ranging from vacuum tubes, to Schottky diodes, and thermionic energy converters. More recently, nanomembrane-based detectors have found applications in high-resolution mass spectrometry measurements in proteomics. Progress in all the aforementioned applications critically depends on discovering materials with effective low surface work functions. We show that a few atomic layer deposition (ALD) cycles of zinc oxide onto suspended diamond nanomembranes, strongly reduces the threshold voltage for the onset of electron field emission which is captured by resonant tunneling from the ZnO layer. Solving the Schroedinger equation, we obtain an electrical field- and thickness-dependent population of the lowest few subbands in the thin ZnO layer, which results in a minimum in the threshold voltage at a thickness of 1.08 nm being in agreement with the experimentally determined value. We conclude that resonant tunneling enables cost-effective ALD coatings that lower the effective work function and enhance field emission from the device.

## Introduction

By definition, the work function of a material is the minimum energy needed to remove an electron from the solid to vacuum in the vicinity of the solid surface. The work function Φ played a central role in Einstein’s seminal explanation of the photoelectric effect, where photons with energies exceeding the work function liberated electrons from the surface of the material^1^. Subsequent studies found that increasing the temperature of the emitter resulted in a significant enhancement in the emission of electrons from the surface. Their relationship between current density *J* and temperature *T* is captured by the Richardson-Dushman (RD) equation *J*(*T*) = *A*_G_*T*^2^exp − Φ/*k*_*B*_*T*^2^, earning its namesake the Nobel prize in 1928. Here, *A*_*G*_ and *k*_*B*_ are the generalized Richardson and the Boltzmann constant, respectively.

In contrast to the thermionic (TH) emission captured by the RD equation, electrons can emit from a cold cathode when applying high electric fields^3^. Electron field emission, which occurs by tunneling through the vacuum barrier, was studied by Fowler and Nordheim^4^. Between the TH and Fowler-Nordheim (FN) regimes, there is an intermediate transition region where emission is affected by both temperature and the electric field^5^. Note, at fields up to 10^8^ V.cm^−1^, the enhanced emission comes from Schottky barrier lowering due to image charges, where the work function is reduced by an applied field *F* according to ${\rm{\Delta }}{\rm{\Phi }}=\sqrt{qF\mathrm{/4}\pi \varepsilon }$ with *q* and *ε* being the electron charge and the vacuum permittivity, respectively. Hence, this electrical field-dependent reduction of the work function allows for an eased extraction of electrons. Nonetheless, lowering the work function Φ further would dramatically increase the emission current *J*(*T, F*).

Low-work-function coatings are crucial in the applications of field emission to enhance the efficiency of thermal-to-electrical energy conversion, refrigeration and cooling as well as photon-assisted thermionic conversion and vacuum nanoelectronics^6–10^. In fact, several theoretical but also experimental research publications showed that adding (ultra-)thin layers of oxides on top of a metal can drastically alter the work function of the compound^11,12^. Indeed, coating of metal cathodes by alkaline earth metal oxides, such as barium oxide, strontium oxide, and calcium oxide to name a few of them, or mixtures of the aforementioned oxides, can reduce the work function of the surface and is already commercially applied in thermionic ‘hot’ field emitters as one may find in laboratory electron guns and electron microscopes^13,14^. Cesium iodide coatings have also been shown to enhance field emission^15^. Recently, it was shown that the work function in large-area monolayer graphene can be lowered by nearly 1 eV through a combination of coating and electrostatic gating^16^.

Another application field is proteomics: Freestanding nanomembranes employing (phonon-assisted) electron field emission have been utilized as detector units in time-of-flight mass spectrometers for protein analysis. It has been shown that membrane-based detectors can extend the accessible protein mass range up to >1 MDa^17–21^. Since the nanomembrane detector utilizes the kinetic energy of the accelerated ions—being the same regardless of its mass—a high sensitivity can be reached, which is largely independent of the ion mass itself and which is not attainable in the standard charge detector configuration nowadays. Optimization of the mechanical and electrical properties of such electron field emitter nanomembranes will lead to further improvement of functionality and sensitivity of the membrane detector unit. Since replacing the membrane’s material is not trivial at all—mechanical properties as well as feasible synthesis processes limiting the spectrum of suitable materials—chemical modification of only the surface, and thus of the work function, might be very attractive.

However, standard physical and chemical vapor phase deposition techniques often suffer from the need of ultra-high vacuum, high deposition temperatures or complex infrastructure of the deposition device. Moreover, precise thickness control in the sub-nm range is quite challenging and requires highly specialized tools. In contrast, atomic layer deposition (ALD) based on sequential gas-solid chemical surface reactions allows for a precise thickness tuning in the Ångström range. The latter fact is caused by the self-limiting nature of the chemical ALD half-reactions. Thus, only moderate technical requirements to vacuum and temperature are needed. Furthermore, the deposition of oxides by physical vapor deposition needs a sensitive control of the oxygen content during the deposition process, whereas the composition and stoichiometry of oxides deposited by ALD is defined by the chemical reaction between a metal ion-containing precursor and an oxidant, e.g. water, ozone, or molecular oxygen^22,23^.

Herein, we utilize ALD to deposit ultra-thin coatings onto nanomembranes to overcome the aforementioned drawbacks of conventional deposition methods. Field emission measurements on nanomembranes reveal a significantly reduced effective work function of the emitting membrane’s surface as a function of ALD cycles. We attribute the observed reduction to resonant tunneling effects in the thin surface layer as predicted by theoretical calculations. In general, our results show that the work function of electron field emitters can be tailor-made when the thickness of a cover layer can be exactly adjusted.

## Experimental Results

**Figure 1:**
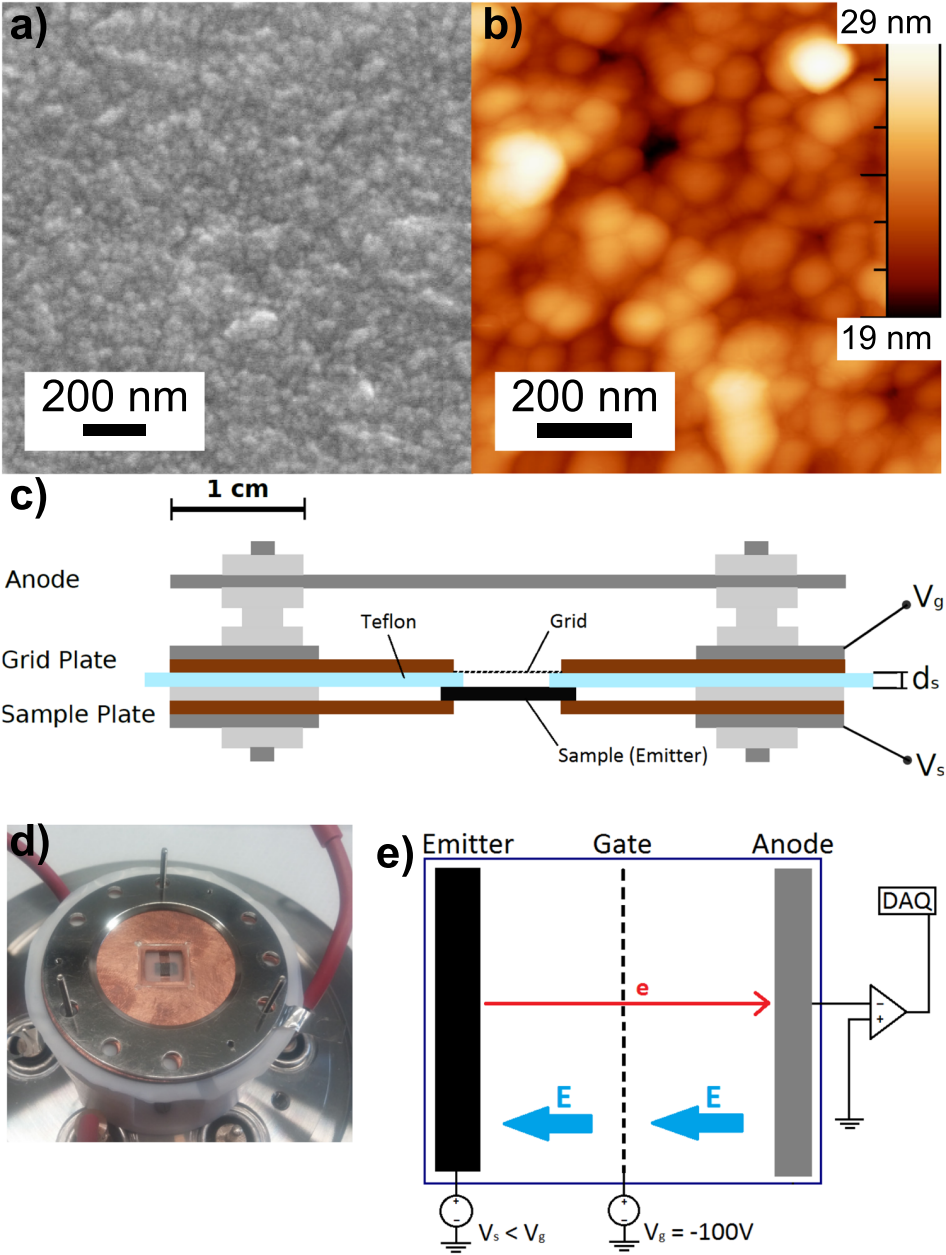
(**a**) SEM and (**b**) AFM image of the top surface of a non-treated diamond nanomembrane. The rough crystalline structure is apparent which eases FE. (**c**) Schematic of the FE measurement setup containing the sample, the grid plate, and the anode. The teflon sheet *d*_*s*_ = 50 µm defines the distance to allow for calculating the applied electric field. (**d**) A photograph of the setup with a nickel grid on top of the teflon distance holder and a test substrate. (**e**) Schematic of the gate configuration and measurement circuit.

A homemade setup is used for field emission test measurements at room temperature (Fig. 1(c,d,e)). It consists of the emitter under test, a metal grid for electron extraction at high fields applied, and an anode at which the extracted electrons are accumulated. When the electric field at the emitter surface is sufficiently high (~1 × 10 ^3^V.μm^−1^), field emission even at room temperature is feasible due to lowering of the vacuum potential barrier which eases the escape of electrons from the material into vacuum via tunneling.

**Figure 2:**
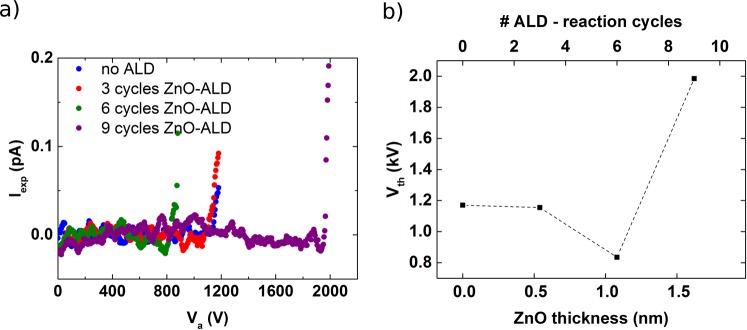
(**a**) Measured current as a function of the applied voltage (I-V) for a pristine diamond membrane and after 3, 6, and 9 cycles of ZnO ALD coating. The data has been smoothen by a simple moving average (n = 10) to guide the eyes. The original data is shown in Supporting Information (Fig. S1). (**b**) Experimentally obtained threshold voltages as a function of number of ALD cycles reveal a non-monotonic behavior with a distinct minimum after 6 cycles corresponding to a deposited ZnO thickness of about 1.08 nm.

Herein, the samples under investigation are suspended, circular diamond nanomembranes with a thickness of 300 nm. The doping concentration of which in combination with their rough surface profile according to the crystal growth (Fig. 1(a,b)) turn them into ideal candidates for electron field emission (FE). In diamond nanomembranes, both thermally-assisted Schottky barrier lowering (SBL) and Fowler-Nordheim (FN) cold field emission mechanisms mainly contribute to the total FE current as a function of the applied electrical field (Fig. S2). We deduce for pristine diamond nanomembranes a FE threshold voltage of 1170 V from the I-V curve (Fig. 2(a) blue curve) which is in good agreement with the literature^24^.

The emission current curve is shifted to lower and higher voltages when the membrane is conformally coated by 3 or 6 and 9 cycles of ZnO ALD, respectively, as depicted in Fig. 2(a). As a consequence, the threshold voltage at which FE starts can be reduced by more than 25%—indicating a significant reduction of the effective work function of the device. After 6 ALD cycles a minimum in the threshold voltage can be observed as shown in Fig. 2(b). Continuing with ALD result in a significant increase of the threshold voltage (9 cycles) and finally exceeding the experimentally accessible voltage range.

## Discussion

Electron emission from resonant quantum structures (such as surface-functionalized multiwall carbon nanotubes, superlattices or ultra-thin coating of wide band gap semiconductor on the emitter) has been reported to occur at fields lower than the conventional FE emission^25–28^. In superlattice emitters, the resonant tunneling is modeled by tuning the hopping parameters to account for structural disorders due to non-uniform bond lengths^29^. The thickness-dependent behavior of threshold electric field has previously been observed on amorphous carbon thin films within a thickness range of 10 nm to 200 nm deposited on *n*-type silicon^30^. In that work, the FE from the silicon substrate is modeled by space-charge-induced band bending mediated by the carbon thin film. Later, a more comprehensive two-step electron tunneling is introduced to calculate the FE in a surface modified Au with WBG polymer using a combination of tunneling probability and attempt frequency^31^. The possibility of local minimum in the threshold field observed here is previously proposed by modeling the FE in surface modified substrates using thin films at varying thickness^32^. There, the FE phenomenon is demonstrated using a self-consistent quantum scheme by considering band bending and electron scattering. In this study, the surface modification of the diamond emitter by deposition of a few ZnO layers leads to the formation of a resonant tunneling layer^33,34^. Confined electron states form in the ZnO layer; these states have energies above the band edge and therefore have stronger tunneling through the surface barrier. To understand the observed initial decrease in the threshold voltage, we implement a numerical model based on resonant FN emission by calculating electron escape rates using the attempt frequencies^26^.

**Figure 3:**
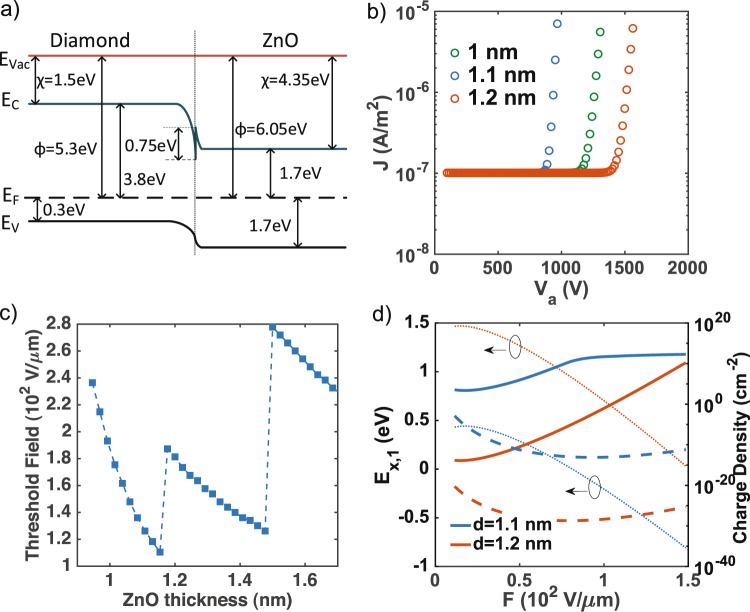
(**a**) Band diagram of a ZnO-coated diamond nanomembrane used for the theoretical modeling. (**b**) Calculated emission current density at applied voltages for different thicknesses of ZnO. A local field enhancement factor *γ* = 8 is used and an offset of 1 × 10^−7^A.m ^−2^ is added to better represent the onset of FE. (**c**) Calculated threshold field in the ZnO film (${F}_{th}$) at different thicknesses of ZnO. (**d**) The charge densities (right axis) of the first (${E}_{(x\mathrm{,1)}}$, solid line) and the second (${E}_{(x\mathrm{,2)}}$, dashed line) subband at the ZnO surface are significantly increased at the thickness at which the emission field discontinuity is observed. This feature can be explained by the downward shift in the conduction band minimum (${E}_{(x\mathrm{,1)}}$) towards Fermi Energy (0 eV) shown as dotted lines (corresponding to left side axis).

The equilibrium band diagram of the $p$-type diamond and ZnO interface constructed by using Anderson’s rule to model the interfacial band alignment in the measurement configuration is shown in Fig. 3(a) ^35,36^. When a strong electric field is applied to the surface of the thin ZnO layer, the band edge forms a triangular potential well with discrete confined energy states. When the field is strong enough, the lowest subband energies at the surface are increased, resulting in resonant cold FE tunneling of electrons. The discrete resonant energies in the potential well can be written as ${E}_{x,n}=U-eFd+{E}_{n}$ where $U$ is the conduction band minimum relative to the Fermi level, $F$ is the electric field in the ZnO film and $d$ is the ZnO film thickness. The energies of discrete subbands in the triangular well, formed inside the thin ZnO surface layer, are given by ${E}_{n}=-\,{a}_{n}{(\frac{{e}^{2}{F}^{2}{\hslash }^{2}}{2m})}^{\frac{1}{3}}$ where $m$ is the electron effective mass in ZnO and ${a}_{n}$ are the zeros of the Airy function. Using this stationary solution to the master rate equation, tunneling is given by $p=\frac{f{{\rm{\Gamma }}}_{L}}{{{\rm{\Gamma }}}_{L}+{{\rm{\Gamma }}}_{R}}$, where $f$ is the equilibrium distribution function of electrons, given by the Fermi-Dirac statistics as $f(E)=\{1+\exp [(E-{E}_{F})/({k}_{B}T)]{\}}^{-1}$.

Despite the fact that there can be many subbands formed in the triangular well, only the first few typically dominate the tunneling because higher subbands are not thermally populated, according to the Fermi-Dirac distribution. The electron escape rates of the left-going (${{\rm{\Gamma }}}_{L}$) and right-going (${{\rm{\Gamma }}}_{R}$) electrons are obtained from barrier transparencies (${D}_{L,R}$) as ${{\rm{\Gamma }}}_{L,R}=v{D}_{L,R}$ using the Wentzel-Kramer-Brillouin (WKB) approximation and attempt frequencies defined as $v=\frac{{E}_{n}}{2\hslash |{a}_{n}{|}^{\frac{3}{2}}}$. Then the charge and the current densities of emitted electrons are calculated by summing over the energies at discrete energy levels as $\sigma =\sum _{n}ep$ and $J=\sum _{n}{{\rm{\Gamma }}}_{R}p$, respectively.

The calculated current densities as a function of ${V}_{a}$ are shown in Fig. 3(b) and mirror the experimental data in Fig. 2(a): We observe an abrupt exponential increase in current density at high voltages—a characteristic of FN field emission—which corresponds to high applied electric fields taking a global field-enhancement factor into account as described previously. Note, the threshold field (${F}_{th}$), which is in our model the strength of the electric field in the ZnO film at which the FN emission overcomes *J* ≈ 1 × 10^−6^ A.m^−2^, shows a non-monotonic dependence on the thickness of the ZnO surface layer (displayed in Fig. 3(c)).

This non-linearity with a sharp minimum threshold value at a specific thickness meets our experimentally measured trend. Moreover, not only the trend but also the minimum position is mirrored. Assuming a negligible growth inhibition at the first cycles, a deposited film thickness of 1.08 nm is expected matching perfectly the calculated thickness of the minimum—compare minimum in Figs 2(b) to 3(c). This minimum non-linearity can be explained by the decrease in the resonant energies ${E}_{x,n}$, thereby increasing the probability of electron emission. Initially, the threshold field decreases with increasing ZnO thickness because the resonant energies of the first resonant subband ${E}_{(x\mathrm{,1)}}$ shifting down and approaching the Fermi level. Hence, the carrier and the charge density in the ZnO layer are increased (solid line in Fig. 3(d)). With increasing thickness a transition into the second resonant subband ${E}_{(x\mathrm{,2)}}$ (dashed line in Fig. 3(d)) follows which then turns the threshold field up before finally converging back to a bulk (large ZnO thickness) value. Increasing the thickness, the corresponding energies of the subbands decrease, allowing the electrons to thermally populate the higher subbands, from which field emission requires higher electric fields. Experimentally, these sharp peaks and valleys (shown in Fig. 3(c)) are often smoothed out by the presence of surface roughness, which broadens the subband energies^37^.

## Conclusions

Our experimental results demonstrate that an ultra-thin coating of few layers of ZnO, deposited by ALD on the surface of a diamond nanomembrane, strongly reduces the threshold voltage for the onset of electron field emission, leading to a minimum at six ALD cycles. The measurements are corroborated by the theory of resonant tunneling in the FN regime. To understand this phenomenon, we calculated the thickness-dependent field emission from confined states in the ZnO layer and observed a decrease in the threshold electric field with increasing thickness, due to the downward shift in the subbands towards the Fermi energy. At a critical thickness of 1.08 nm, corresponding to roughly 6 ALD cycles, there is a cross-over to the second subband, which is far from the Fermi energy, and a reversal of the downward trend. This trend continues as thickness is increased and higher subbands are filled. Our work uncovers the possibility to significantly lower the threshold value of electron field emitter devices by coating them with an ultra-thin layer of wide-bandgap ZnO.

Indeed and in terms of generality, the ultra-thin deposited coating material on top of the emitter can be in fact any wide band gap material; the optimal thickness has to be finely tuned depending on the physical and chemical properties of the substrate and the emitter, such as band bending/alignment, Fermi level position, and stability to name a few of them. In detail at the example of thin titania (TiO_2_), the heavier effective mass of the charge carriers (compared to ZnO) would result in a reduced current density, even though it has smaller bandgap as well as smaller work function (3.4 eV and 5.2 eV, respectively^38^); the larger dielectric constant would increase the threshold voltage required for field emission. Note, ALD as a versatile standard tool in semiconductor industry is perfectly suited to allow for large scale and thus economically feasible production of tailor-made field emitters with low threshold field for electron field emission because of its sub-nm precision, its unique conformality and homogeneity, as well as the variety of materials which can be deposited. With respect to applications, the herein presented modified nanomembranes might act a launching platform for stable, user-friendly membrane detectors in proteomics with a much higher sensitivity in the large protein mass range than conventional detectors. ALD coatings to prepare low work function devices may also increase the efficiency of thermionic heat-to-electricity conversion and refrigeration, photon-enhanced solar conversion, and nanoelectronics cooling.

## Experimental Methods

All field emission measurements are performed under high vacuum of at least 2 × 10^−7^ mbar in order to avoid undesirable scattering of the field extracted electrons and electrical breakdown in the assembly. A high negative sample potential ${V}_{{\rm{a}}}=|{V}_{{\rm{s}}}-{V}_{{\rm{g}}}|$ can be achieved between emitter and gate, which is a nickel mesh with 59 lpc, by applying a tunable sample voltage ${V}_{s} > -\,2500\,V$ as well as a grid voltage ${V}_{g}=-\,100\,V$. The latter is kept constant for all measurements. Between the two electrodes an insulating teflon sheet defines the distance ${d}_{s}\,=\,50\,\mu m$.

Electrons released by field emission from the sample under test are accelerated towards the anode—a polished Ni sheet—schematically shown in Fig. 1(e), at which being collected and guided towards the read-out consisting of a transimpedance amplifier for signal magnification and the data acquisition system (DAQ). The measurable output current ${I}_{{\rm{out}}}$ is taken from integrative measurements (integration time 2 s with about 2 × 10^6^ data points) at various applied voltages in order to obtain the $I$-$V$ characteristic of the investigated nanomembrane. Specifically, each current measurement is taken from eight circular nanomembranes with ${A}_{{\rm{e}}{\rm{f}}{\rm{f}}}=125\,{\mu {\rm{m}}}^{2}/{\rm{m}}{\rm{e}}{\rm{m}}{\rm{b}}{\rm{r}}{\rm{a}}{\rm{n}}{\rm{e}}$ and is reduced by a global current offset and a linear increase in current with applied voltage caused by the amplifier offset and leakage currents in the assembly, respectively. The threshold voltage ${V}_{{\rm{th}}}$ of FE is defined as the value at which the measured current overcomes a 5σ limit from the data mean.

The p-doped diamond nanomembranes are coated by ALD of ZnO which is performed in a homemade reactor at 150 °C utilizing diethylzinc and water (both kept at room temperature) as precursors with a constant nitrogen flow of about 30 sccm. The pulse, exposure, and purge time for both precursors are set to 200 ms, 10 s, and 60 s, respectively. The growth per cycle of the ZnO process used herein has been determined to 1.8 Å by spectroscopic ellipsometry in a reference process of 200 cycles on a plain silicon substrate being in good agreement with reported literature values. Note, taking a c-axis value of 0.522 nm for thin zinc oxide films deposited by ALD into account, 3 cycles would correspond to about one monolayer of ZnO.

## Supplementary information

Supporting Information
