# Supplementary material for: Resonant Tunneling Induced Enhancement of Electron Field Emission by Ultra-Thin Coatings
Source: Sci Rep. 2019 May 2;9:6840. doi: 10.1038/s41598-019-43149-y (PMC6497713; doi:10.1038/s41598-019-43149-y)
Supplement: Supplementary file 1 — Supporting Information [file 41598_2019_43149_MOESM1_ESM.pdf]

Supporting Information to:

Resonant Tunneling Induced Enhancement of Electron Field Emission by Ultra-Thin Coatings

Christian Henkel[1] , Robert Zierold[1] , Adithya Kommini[2] , Stefanie Haugg[1] , Chris Thomason[1] , Zlatan Aksamija[2] , and Robert H. Blick[1,3]*

1 Center for Hybrid Nanostructures (CHyN), Universität Hamburg, Luruper Chaussee 149, 22761 Hamburg, Germany

2 Electrical and Computer Engineering, University of Massachusetts, 100 Natural Resources Road, Amherst, Massachusetts 01003-9292, United States

3 Materials Science and Engineering, University of Wisconsin-Madison, 1550 University Avenue, Madison, WI53706, USA

* rblick@chyn.uni-hamburg.de

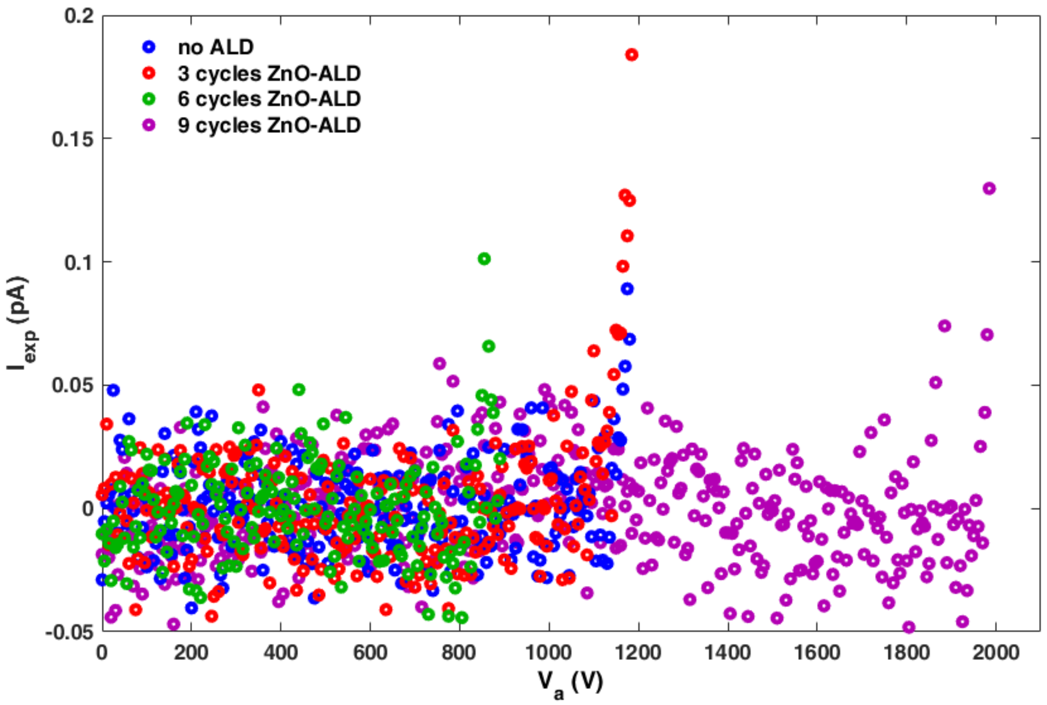

Figure S1 Original data: Measured current as a function of the applied voltage (I-V) for a pristine diamond membrane and after 3, 6, and 9 cycles of ZnO ALD coating.

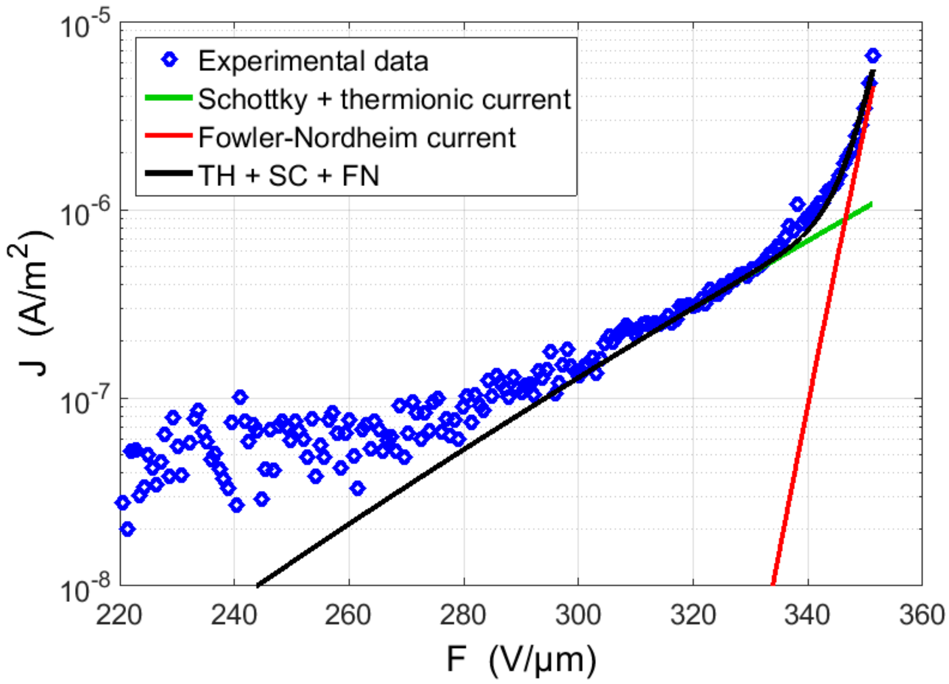

Figure S2 FE analysis of the diamond nanomembranes via thermionic (TH), Schottky (SC), and Fowler-Nordheim (FN) emission mechanism. The superimposed current density from all theoretically fitted mechanisms (black line) matches the experimentally obtained data from the FE measurement (blue circles) in the electric field region F > 300 V/µm.

The apparent local electric field is defined as $F=\gamma V_a/d_s$ with a field enhancement factor γ which has its origin in geometrical features on the emitter surface and leads to a local increase of the externally applied electric field, V_a the applied voltage and d_s the distance between grid and sample surface. Since diamond membranes are slightly rippled because of the intrinsic stress, we assumed an additional reduction of the 50 µm Teflon sheet by about 24 µm (Supplementary Material [1]). By fitting the experimentally derived I-V-curve of a pristine diamond nanomembrane—with a sum of the different current density distributions according to literature [1] —we determined γ to be 5.15, which is caused by the microstructure of the membrane's surface (see Fig.1 (b)) leading to a concentration of the electrical field. Note, a thin surface coating may change the surface morphology and the residual mechanical stress of the free-standing membranes and thus might alter the gamma value slightly.

[1] Kim, H., Park, J., Aksamija, Z., Arbulu, M. & Blick, R. H. Ultrananocrystalline Diamond Membranes for Detection of High-Mass Proteins. Physical Review Applied 6, 1–7 (2016).
